# Achievable Rate Region under Linear Beamforming for Dual-Hop Multiple-Access Relay Network

**DOI:** 10.3390/e20080547

**Published:** 2018-07-24

**Authors:** Guiguo Feng, Wangmei Guo, Binyue Liu

**Affiliations:** 1 The State Key Laboratory of Integrated Services Network, Xidian University, Xi’an 710071, China; 2 Bell Labs., Alcatel-Lucent, Shanghai 201206, China

**Keywords:** beamforming, multiple-access relay network (MARN), achievable rate region, semi-definite relaxation, semi-definite programming

## Abstract

Consider a network consisting of two independent single-antenna sources, a single-antenna destination and a helping multiple-antenna relay. This network is called a dual-hop multiple access relay network (MARN). In this network, sources transmit to the relay simultaneously in the first time slot. The relay retransmits the received sum-signal to the destination using a linear beamforming scheme in the second time slot. In this paper, we characterize the achievable rate region of MARN under linear beamforming. The achievable rate region characterization problem is first transformed to an equivalent “corner point” optimization problem with respect to linear beamforming matrix at the relay. Then, we present an efficient algorithm to solve it via only semi-definite programming (SDP). We further derive the mathematical close-forms of the maximum individual rates and the sum-rate. Finally, numerical results demonstrate the performance of the proposed schemes.

## 1. Introduction

Wireless networks today are facing with challenges of high demands of reliable transmission and throughput while reducing signal interference. Relay-based cooperative networks are proposed to overcome these challenges and the essence here is to design relay strategies among sources and relay nodes. Compared with others like decode-forward (DF) and compress-forward (CF), amplified-forward (AF) has advantages of simple implementation and low relaying cost and is thus preferred in designing cooperative networks [1,2]. In fact, it has been shown optimal in some cases [3].

One popular variant of AF is linear beamforming. It achieves high transmission rate by generating pencil beams to concentrate signals in a narrow direction towards intended receivers, and therefore significantly reduces interference from omni-directional antenna transmissions. For this reason, linear beamforming is widely applied in wireless relay networks as a promising relaying strategy [4,5]. The objective of linear beamforming design is to find the optimal beamforming vector that achieves maximum end-to-end data rate under power constraints, either individually or by sum-power [6,7,8]. Depending on multiple antennas at relay, the objective becomes to find a beamforming matrix in multiple-input-multiple-output (MIMO) systems [9,10,11]. In the latter case, it is actually to solve a matrix-monotonic optimization problem.

Another approach to avoid collision in wireless half-duplex mode is analog network coding (ANC). In [12], Katti et al. proposes an analog network coding (ANC) relaying scheme, which allows relay nodes to receive the sum-signal from multiple sources in one time slot. When we have two separate sources, we can utilize ANC to combine the process of multiple signals and beamforming to address the capacity. Such a combination of ANC and AF [13] has been shown to improve the performance.

In [14], the authors make the first attempt to investigate the error rate and the power allocation of a MARN with ANC uninvolved with the system capacity. However, the capacity is more important in many cases. There have been several attempts to achieve high information rate by finding optimal beamforming in MARN. The pioneer work [15] fully characterizes the complete achievable rate region of a dual-hop MARN where linear beamforming is employed under the sum power constraint. To obtain the optimal linear beamforming vector, the authors propose weighted sum-rate maximization approach. Unfortunately, when power is constrained individually rather than in a sum, such approach is hard to solve [16] for its prohibitive computational complexity. A new and effective method is proposed in [17] to find the optimal linear beamforming vector for the same MARN under an individual power constraint. Note that existing work assumes that the relays have only one antenna. To the best of our knowledge, the achievable rate region for a multi-antenna relay is still unknown.

In this paper, we investigate communications on MARN with two independent source nodes, one destination node and one multi-antenna relay. This network model captures various wireless systems like wireless monitoring system (WMS), in which multiple monitoring terminals transmit messages to a monitoring center through a multi-antenna relay station for remote monitoring under the sum-power constraint. Both ANC and beamforming are applied at the relay node.

Our main contribution of this paper is to propose an efficient scheme to characterize the achievable rate region and its corresponding linear beamforming matrix. Specifically, we give a families of corner point (CP) optimization formulations to determine the achievable rate region by solving a series of CP non-convex optimization problems. We then show how to transform them to semi-positive definite (SDP) convex problems. To lower the computational burden of solving SDP, we propose an optimal structure of linear beamforming matrix such that the number of independent variables in linear beamforming matrix is reduced to a small constant. Finally, we derive the mathematical close-forms of the maximum individual rates and the sum-rate. Our scheme has the following advantages:

- High transmission rate: Beamforming can suppress the inference from antennas in relay for its targeted nature. ANC can fully utilize the inference from different users rather than avoid it to improve the transmission signal-to-noise ratio (SNR). Based on the simulation results, we find that our scheme can obtain a higher transmission rate at the same cost.
- Low computational complexity: we propose an optimal structure of an AF matrix employing singular-value decomposition, and reduce the number of design variables in the relay beamforming matrix from $K^{2}$ to $r^{2}$, r∈(1,2,3). Then, the number of variable parameters in SDP will decrease significantly.

The rest of this paper is organized as follows. The network model and problem statement are presented in Section 2. The method to design the ANC beamforming matrix is presented in Section 3. The mathematical closed-forms of the maximum individual rates and the sum rate are derived in Section 4. Then, the simulations are given in Section 5, and all of the detailed proofs are arranged in appendixes.

***Notation***: Scalars are denoted by lower-case letters, e.g., *x*, and bold-face lower-case letters are used for column vectors, e.g., x, and bold-face upper-case letters for matrices, e.g., X and let $X=[x_{1},x_{2},\cdots ,x_{n}]$. In addition, tr(·), det(·), ${\left(\cdot \right)}^{*}$, ${\left(\cdot \right)}^{T}$, ${\left(\cdot \right)}^{†}$ and ${\left(\cdot \right)}^{-1}$ denote the trace, determinant, conjugate, transpose, Hermitian transpose and inverse matrix, respectively. $\mathrm{blkdiag}(X_{1},\cdots ,X_{n})$ denotes a block-diagonal square matrix with $X_{1},\cdots ,X_{n}$ as the diagonal elements, and $\mathrm{vec}\left(X\right)\;=\;{\left[x_{1}^{T},x_{2}^{T},\cdots ,x_{n}^{T}\right]}^{T}$. ⊗ denotes the Kronecker product. ||·|| denotes the Euclidean norm. $I_{n}$ is the *n* identity matrix. $E\left[\cdot \right]$ is the expectation operation. log(·) denotes the logarithm in the base 2.

## 2. Network Model

**Figure 1:**
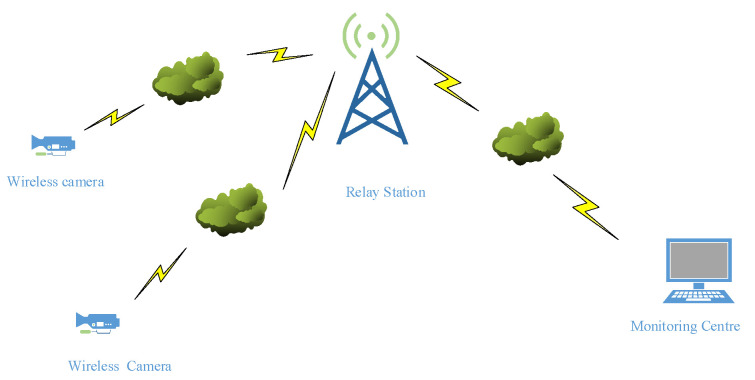
Wireless monitoring system.

**Figure 2:**
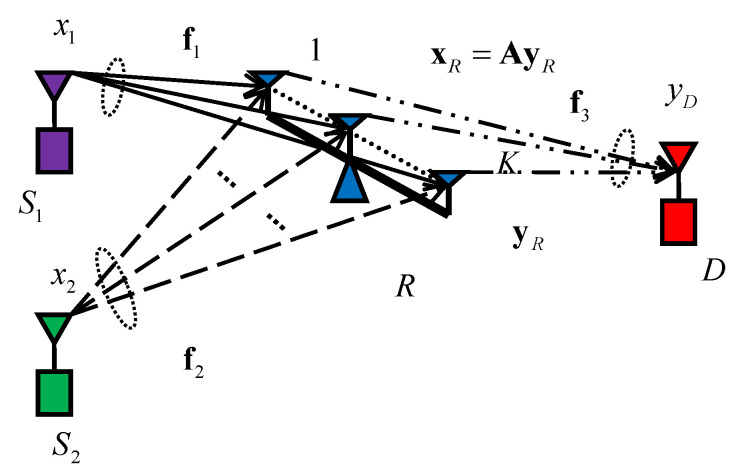
Network model.

Consider a wireless monitoring system in Figure 1. In this paper, the direct links between the two wireless cameras and monitoring center are ignored since the monitor is far away the cameras. In addition, the destination receives the signals in maximal ratio combining (MRC). The system SNR is the sum of SNRs in all links. Apparently, the SNRs in these direct links are independent of the linear beamforming matrix. It does not affect the design of beamforming. This actual transmission model can be transformed mathematically as a MARN shown in Figure 2.

The MARN consists of two single antenna sources, $S_{1}$ and $S_{2}$, a helping *K* antennas relay *R*, and a single antenna destination *D*. All of the channels are assumed flat-fading over a common narrow-band. The relay works in half-duplex mode and there is no direct link from $S_{1}$ and $S_{2}$ to *D*. For a Gaussian multiple access channel, time division multiple access (TDMA), frequency division multiple access (FDMA), and code division multiple access (CDMA) are the general multiple access modes. In TDMA mode, the sources use non-overlapping time periods to complete the transmission. Thus, the receiver can separate signals according to different time periods. In FDMA mode, the sources transmit signals simultaneously in the mutually disjoint frequency bands. Then, the receiver can separate signals according to different frequency bands. In the CDMA mode, the sources transmit the signals using different codes simultaneously. Thus, the receiver decodes one by one to separate the signals. According to [18], the multiple access channel can achieve a larger rate region in CDMA mode.

In this paper, the two sources will complete the communication in CDMA. Assume that the sources $S_{1}$ and $S_{2}$ independently generates the $2^{nr_{1}}$ and $2^{nr_{2}}$ codewords in the rates $r_{1}$ and $r_{2}$ with the block-length *n*. Note, in this paper, $r_{1}$ and $r_{2}$ represent the information rates rather than the data rates or frame rates. The code symbols $x_{1}∼\mathrm{CN}\left(0,P_{1}\right)$ and $x_{2}∼\mathrm{CN}\left(0,P_{2}\right)$. $P_{1}$ and $P_{2}$ denote the transmit powers of $S_{1}$ and $S_{2}$. In practice, $S_{1}$ and $S_{2}$ transmit the codewords $x_{1}$ and $x_{2}$ with the block-length *n* to the relay simultaneously. The destination performs decoding after receiving $y_{D}$ with the block-length *n*. The method of separating these two signals at the destination through decoding will be described later. In this case, the frame rates of the two sources are the same. Therefore, we can perform ANC at the relay without affecting decoding. Since the lengths of the two codewords from $S_{1}$ and $S_{2}$ are the same, we can consider this mode through discussing the single symbol transmission. During the first time slot, both $S_{1}$ and $S_{2}$ transmit simultaneously to *R*, which uses a linear beamforming scheme to retransmit the received sum signal with noise to *D* during the second time slot. It is also assumed that perfect synchronization are established among $S_{1}$ and $S_{2}$ prior to data transmission. The baseband signal received at *R* in the first time slot is expressed as

(1) $$y_{R}=f_{1}x_{1}+f_{2}x_{2}+z_{R},$$

where $y_{R}\in C^{K\times 1}$ is the signal vector received at *R*; $f_{1}\in C^{K\times 1}$ and $f_{2}\in C^{K\times 1}$ represent the channel vectors from $S_{1}$ to *R* and from $S_{2}$ to *R*, respectively, which are assumed constant during the transmission. $z_{R}\in C^{K\times 1}$ is noise vector at the relay, and without loss of generality (w.l.o.g.), $z_{R}∼\mathrm{CN}\left(0,I_{K}\right)$. For ANC is exploited in the antennas of relay, the signals from $S_{1}$ and $S_{2}$ are summed in the antennas. Upon the *K* sum-signals, the relay processes them employing a linear beamforming matrix A, and then retransmits it to *D* during the second time slot. Mathematically, the signals vector retransmitted at *R* can be concisely represented as

(2) $$x_{R}={\mathrm{Ay}}_{R},$$

where $x_{R}\in C^{K\times 1}$ is the signal vector retransmitted at *R*, and $A\in C^{K\times K}$ is the beamforming matrix.

The relay has a power budget $P_{R,\max}$. Thus, the signal vector retransmitted at *R* should satisfy the following constraint:

(3) $$E[|x_{R}{|}^{2}]=||{\mathrm{Af}}_{1}{\left|\right|}^{2}P_{1}+\left|\right|{\mathrm{Af}}_{2}{\left|\right|}^{2}P_{2}+\mathrm{tr}\left({\mathrm{AA}}^{†}\right)\leq P_{R,\max}.$$

We use Ω={A|AsatisfiesEquation(3)} to denote the set of all beamforming matrices satisfying the relay power constraint. As a result, given a beamforming matrix A, the signal received at *D* can be expressed as

(4) $$y_{D}=f_{3}^{T}{\mathrm{Af}}_{1}x_{1}+f_{3}^{T}{\mathrm{Af}}_{2}x_{2}+f_{3}^{T}{\mathrm{Az}}_{R}+z_{D},$$

where $z_{D}∼\mathrm{CN}\left(0,1\right)$ is the noise at destination, and $f_{3}\in C^{K\times 1}$ denotes the channel vector from *R* to *D*. We assume that perfect channel state information (CSI) has been collected at *R* prior to transmission.

From Equation (4), the dual-hop MARN with linear beamforming can be considered as a conventional Gaussian multiple-access channel (MAC) on matrix A as follows:

(5) $$\begin{array}{c} y_{D}\left(A\right)=x_{\mathrm{eq},1}\left(A\right)+x_{\mathrm{eq},2}\left(A\right)+z_{\mathrm{eq}}\left(A\right), \end{array}$$

where $x_{\mathrm{eq},1}\left(A\right)=f_{3}^{T}{\mathrm{Af}}_{1}x_{1}$ and $x_{\mathrm{eq},2}\left(A\right)=f_{3}^{T}{\mathrm{Af}}_{2}x_{2}$ are the information symbols of the two equivalent sources and $z_{\mathrm{eq}}\left(A\right)=f_{3}^{T}{\mathrm{Az}}_{R}+z_{D}$ is the equivalent Gaussian noise drawn according to $CN(0,||f_{3}^{T}A|{|}^{2}+1)$. To distinguish them, we denote the former one as MAC(A). The capacity region of a Gaussian MAC $y=x_{1}+x_{2}+z$ can be found in ([18], Section 14.3). According to the capacity region of the Gaussian MAC, the achievable rate region of MAC(A) is denoted by R(A) given as follows

(6) $$\left.R\left(A\right)=\left\{\begin{array}{cc} \left(r_{1}\left(A\right),r_{2}\left(A\right)\right):r_{1}\left(A\right) & \leq \frac{1}{2}\log\left(1+\frac{|f_{3}^{T}Af_{1}{|}^{2}P_{1}}{\left|\right|f_{3}^{T}A{\left|\right|}^{2}+1}\right)\\ r_{2}\left(A\right) & \leq \frac{1}{2}\log\left(1+\frac{|f_{3}^{T}Af_{2}{|}^{2}P_{2}}{\left|\right|f_{3}^{T}A{\left|\right|}^{2}+1}\right)\\ r_{1}\left(A\right)+r_{2}\left(A\right) & \leq \frac{1}{2}\log\left(1+\frac{|f_{3}^{T}Af_{1}{|}^{2}P_{1}+{\left|f_{3}^{T}Af_{2}\right|}^{2}P_{2}}{\left|\right|f_{3}^{T}A{\left|\right|}^{2}+1}\right) \end{array}\right.\right\}.$$

For notation brevity, we denote $C_{1}\left(A\right)=\frac{1}{2}\log\left(1+\frac{|f_{3}^{T}Af_{1}{|}^{2}P_{1}}{\left|\right|f_{3}^{T}A{\left|\right|}^{2}+1}\right)$, $C_{2}\left(A\right)=\frac{1}{2}\log\left(1+\frac{|f_{3}^{T}Af_{2}{|}^{2}P_{2}}{\left|\right|f_{3}^{T}A{\left|\right|}^{2}+1}\right)$, $C_{\mathrm{sum}}\left(A\right)=\frac{1}{2}\log\left(1+\frac{|f_{3}^{T}Af_{1}{|}^{2}P_{1}+{\left|f_{3}^{T}Af_{2}\right|}^{2}P_{2}}{\left|\right|f_{3}^{T}A{\left|\right|}^{2}+1}\right)$ and the union of the achievable rate sets R(A)’s by $R\;=\;\cup_{A\in \Omega }R\left(A\right)$. By the time-sharing technique, the achievable rate region of an MARN is given by cvx(R), where cvx(·) represents the convex hull of a set.

In information theory, the hypothesis that the decoder at the destination knows the codebooks of the source $S_{1}$ and the $S_{2}$ is practical. First, the decoder amplifies the all $2^{nr_{1}}$ codewords $x_{1}$ with $f_{3}^{T}{\mathrm{Af}}_{1}$ to obtain $2^{nr_{1}}$ sequences ${\hat{x}}_{1}$ with the length *n* not changing their corresponding messages. Similarly, the decoder can obtain $2^{nr_{2}}$ new sequences ${\hat{x}}_{2}$ employing $f_{3}^{T}{\mathrm{Af}}_{2}$. Second, the decoder selects the sequence combination $\left({\hat{x}}_{1},{\hat{x}}_{2}\right)$ with the smallest Euclidean distance from y among all sequence combinations. Then, the decoder can determine the codeword combination $\left(x_{1},x_{2}\right)$ transmitted by the sources $S_{1}$ and $S_{2}$ corresponding the sequence combination $\left({\hat{x}}_{1},{\hat{x}}_{2}\right)$. In this paper, we do not discuss the specific decoding scheme in practice. We only cite the conclusion of Gaussian multiple access channel capacity region in information theory.

It should be pointed out that this achievable rate region is obtained in CDMA mode with the synchronization condition. Unfortunately, synchronization is very difficult to achieve even in the small distance. In addition, the paper [19] pointed out that lack of synchronization can not reduce the capacity region for multiple access channel when the block lengths of the codes are long compared to the delay. Thus, in this paper, if the synchronization condition cannot be satisfied, as long as the packet length of the codes is long compared to the delay, the achievable rate region of the MARN remains unchanged.

From Equation (6), we can determine the outer bound of the achievable rate region R(A) by maximizing $C_{1}\left(A\right)$, $C_{2}\left(A\right)$ and $C_{\mathrm{sum}}\left(A\right)$ under the condition A∈Ω, respectively. We will obtain the maximum values $\max_{A\in \Omega }\;C_{1}\left(A\right)$, $\max_{A\in \Omega }\;C_{2}\left(A\right)$ and $\max_{A\in \Omega }\;C_{\mathrm{sum}}\left(A\right)$ and the corresponding optimal beamforming matrices $A_{\mathrm{opt},1}$, $A_{\mathrm{opt},2}$ and $A_{\mathrm{opt},\mathrm{sum}}$. Note that they are not unique. Usually, there exists the situation as follows:

(7) $$\left\{\begin{array}{c} C_{1}\left(A_{\mathrm{opt},2}\right)<C_{1}\left(A_{\mathrm{opt},1}\right),C_{1}\left(A_{\mathrm{opt},\mathrm{sum}}\right)<C_{1}\left(A_{\mathrm{opt},1}\right),\\ C_{2}\left(A_{\mathrm{opt},1}\right)<C_{2}\left(A_{\mathrm{opt},2}\right),C_{2}\left(A_{\mathrm{opt},\mathrm{sum}}\right)<C_{2}\left(A_{\mathrm{opt},2}\right),\\ C_{\mathrm{sum}}\left(A_{\mathrm{opt},1}\right)<C_{1}\left(A_{\mathrm{opt},\mathrm{sum}}\right),C_{\mathrm{sum}}\left(A_{\mathrm{opt},2}\right)<C_{\mathrm{sum}}\left(A_{\mathrm{opt},\mathrm{sum}}\right). \end{array}\right.$$

**Figure 3:**
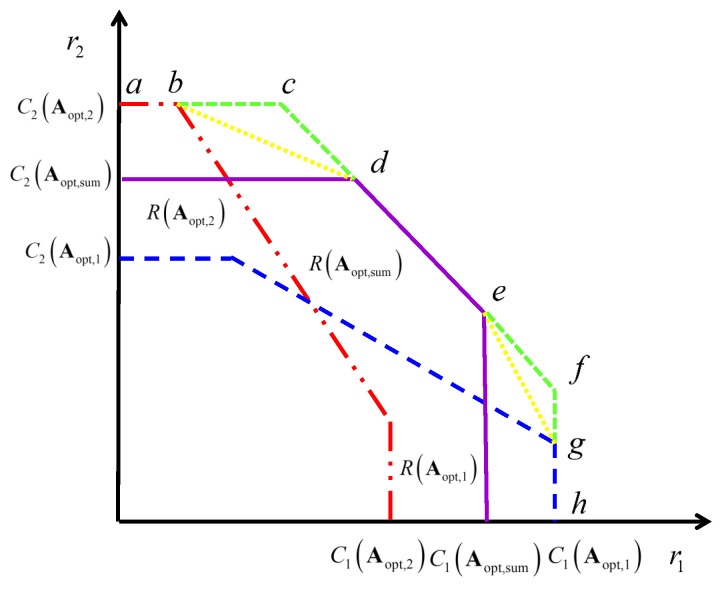
Relationship of the Achievable Rate Regions.

We will provide the relationship of the achievable rate regions in Figure 3. $R(A_{\mathrm{opt},1})$ is in the blue area, $R(A_{\mathrm{opt},2})$ is in the red area, and $R(A_{\mathrm{opt},\mathrm{sum}})$ is in the violet area.

From Figure 3, it is easy to obtain the outer bound of the rate region consisting of the polyline a→c→f→h, employing these three values $C_{1}\left(A_{\mathrm{opt},1}\right)$, $C_{2}\left(A_{\mathrm{opt},2}\right)$, $C_{\mathrm{sum}}\left(A_{\mathrm{opt},\mathrm{sum}}\right)$. However, according to the relationship of $R(A_{\mathrm{opt},1})$, $R(A_{\mathrm{opt},2})$, $R(A_{\mathrm{opt},\mathrm{sum}})$, we can not determine the beamforming matrices corresponding to the dotted line subregions b→c→d and e→f→g in Figure 3. Therefore, it is unknownwhether these two subregions are achievable. Additionally, using time-sharing, we can obtain the straight line b→d of the “upper corner” of regions $R(A_{\mathrm{opt},2})$ and $R(A_{\mathrm{opt},\mathrm{sum}})$ and e→g of the “low corner” of regions $R(A_{\mathrm{opt},1})$ and $R(A_{\mathrm{opt},\mathrm{sum}})$. Obviously, the inner bound of the rate region with polyline a→b→d→e→g→h is achievable. Thus, these two subregions ▵bcd and ▵efg between the inner bound and outer bound are unknown in Figure 3. We will propose another new method to determine the achievable rate region and the corresponding beamforming matrices in Section 3, and discuss whether this outer bound is tight in Section 4.

## 3. Design of ANC Encoding Matrix

**Figure 4:**
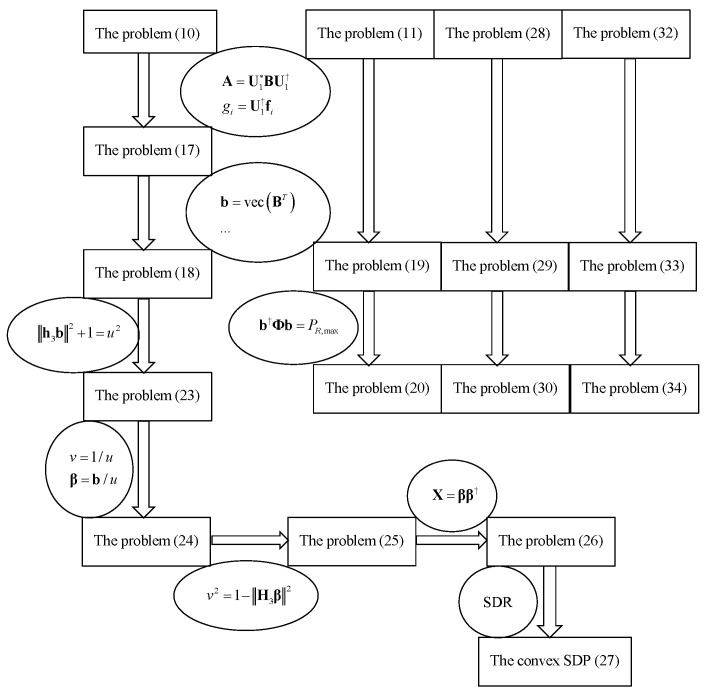
Relationship of the problems.

In this section, we show that the problem of characterizing R(A) can be formulated as an equivalent non-convex CP optimization problem. With several transformation tricks developed in this study, we show that the CP optimization problem can be efficiently solved via a semi-definite programming (SDP)-based approach. In Section 3 and Section 4, the paper deals with many equivalent transformations of optimization problems, which are tedious. We use the following Figure 4 to illustrate the relationships of these series of optimization problems so that readers can understand easily.

### 3.1. Corner-Point Optimization Problem

We consider two special achievable rate pairs in R(A). Using the successive cancelation decoding scheme with different decoding orders, the following rate pairs can be achieved:

(8) $$\begin{array}{c} \left\{\begin{array}{c} R_{1}^{\mathrm{up}}\left(A\right)=\frac{1}{2}\log\left(1+\frac{|f_{3}^{T}Af_{1}{|}^{2}P_{1}}{|f_{3}^{T}Af_{2}{|}^{2}P_{2}+\left|\right|f_{3}^{T}A{\left|\right|}^{2}+1}\right),\\ R_{2}^{\mathrm{up}}\left(A\right)=\frac{1}{2}\log\left(1+\frac{|f_{3}^{T}Af_{2}{|}^{2}P_{2}}{\left|\right|f_{3}^{T}A{\left|\right|}^{2}+1}\right), \end{array}\right. \end{array}$$

(9) $$\begin{array}{c} \left\{\begin{array}{c} R_{1}^{\mathrm{low}}\left(A\right)=\frac{1}{2}\log\left(1+\frac{|f_{3}^{T}Af_{1}{|}^{2}P_{1}}{\left|\right|f_{3}^{T}A{\left|\right|}^{2}+1}\right),\\ R_{2}^{\mathrm{low}}\left(A\right)=\frac{1}{2}\log\left(1+\frac{|f_{3}^{T}Af_{2}{|}^{2}P_{2}}{|f_{3}^{T}Af_{1}{|}^{2}P_{1}+\left|\right|f_{3}^{T}A{\left|\right|}^{2}+1}\right). \end{array}\right. \end{array}$$

It is easy to verify that $R_{1}^{\mathrm{up}}\left(A\right)+R_{2}^{\mathrm{up}}\left(A\right)=C_{\mathrm{sum}}\left(A\right)$, $R_{1}^{\mathrm{low}}\left(A\right)+R_{2}^{\mathrm{low}}\left(A\right)=C_{\mathrm{sum}}\left(A\right)$. It is observed that $R_{2}^{\mathrm{up}}\left(A\right)=C_{2}\left(A\right)$, $R_{1}^{\mathrm{low}}\left(A\right)=C_{1}\left(A\right)$. We call these two points $\left(R_{1}^{\mathrm{up}}\left(A\right),R_{2}^{\mathrm{up}}\left(A\right)\right)$ and $\left(R_{1}^{\mathrm{low}}\left(A\right),R_{2}^{\mathrm{low}}\left(A\right)\right)$ as the “upper-diagonal” and “lower-diagonal” corner points of R(A), respectively.

Without loss of generality, we only consider the “upper-diagonal” corner point (8), similar to the “lower-diagonal” corner point. According to (8), we set up a problem of maximizing the transmission rate $R_{2}^{\mathrm{up}}\left(A\right)$ under the relay power constraints (3). It is necessary to add a constraint that the transmission rate $R_{1}^{\mathrm{up}}\left(A\right)$ is no less than a desired value $r_{1}$ to ensure the transmission rate of user 1 is $r_{1}$ at least. Combining the fact that log(·) is an increasing function, the above-mentioned maximization problem can be formulated as follows, which is referred to as CP optimization problems:

(10) $$\begin{array}{cc} \max_{A}\;\; & \frac{{\left|f_{3}^{T}{\mathrm{Af}}_{2}\right|}^{2}P_{2}}{\left|\right|f_{3}^{T}A{\left|\right|}^{2}+1},\\ s.t.\;\; & \frac{{\left|f_{3}^{T}{\mathrm{Af}}_{1}\right|}^{2}P_{1}}{{\left|f_{3}^{T}{\mathrm{Af}}_{2}\right|}^{2}P_{2}+\left|\right|f_{3}^{T}A{\left|\right|}^{2}+1}\geq \gamma_{1}\\ & \left|\right|{\mathrm{Af}}_{1}{\left|\right|}^{2}P_{1}+\left|\right|{\mathrm{Af}}_{2}{\left|\right|}^{2}P_{2}+\mathrm{tr}\left({\mathrm{AA}}^{†}\right)\leq P_{R,\max}, \end{array}$$

where $\gamma_{1}=2^{2r_{1}}-1$ is the equivalent SNR constraint. We denote the optimal solution of the problem (10) as $A_{o}\left(r_{1}\right)$. For notation brevity, we denote $R(A_{o}\left(r_{1}\right))$ by $R(r_{1})$. Furthermore, the maximum possible value of $r_{1}$ can be determined via solving the following problem:

(11) $$\begin{array}{cc} \max_{A}\;\; & \frac{{\left|f_{3}^{T}{\mathrm{Af}}_{1}\right|}^{2}P_{1}}{{\left|f_{3}^{T}{\mathrm{Af}}_{2}\right|}^{2}P_{2}+\left|\right|f_{3}^{T}A{\left|\right|}^{2}+1},\\ s.t.\;\;\;\; & \left|\right|{\mathrm{Af}}_{1}{\left|\right|}^{2}P_{1}+\left|\right|{\mathrm{Af}}_{2}{\left|\right|}^{2}P_{2}+\mathrm{tr}\left({\mathrm{AA}}^{†}\right)\leq P_{R,\max}. \end{array}$$

The maximum objective value is denoted by $\gamma_{1,\max}$, which can be easily obtained using the approach proposed in [6]. Then, we have $r_{1,\max}=\frac{1}{2}\log\left(1+\gamma_{1,\max}\right)$.

**Theorem** **1.** *For a rate pair $(r_{1},r_{2})\in R$, then, $\left(r_{1},r_{2}\right)\in R\left(r_{1}\right)$.*

**Proof.**  See in Appendix A. □

From Theorem 1, we can obtain a straightforward corollary shown below.

**Corollary** **1.** *The achievable rate region of a MARN cvx(R) is equal to $\mathrm{cvx}(\cup_{r_{1}\in \left[0,r_{1,\max}\right]}R\left(r_{1}\right)).$*

According to Theorem 1 and Corollary 1, we can characterize the achievable rate region of the MARN by the following method:

1. Solve problem (11) to obtain $\gamma_{1,\max}$.
2. Divide the interval $\left[0,\gamma_{1,\max}\right]$ into some sufficiently small intervals, such that the length of the small interval equals ε.
3. Solve problem (10) according to the dividing point $\gamma_{1}$ and update $\gamma_{1}=\gamma_{1}+\varepsilon$.
4. Record the optimal value and corresponding solution of the problem (10), and repeat step 3 until $\gamma_{1}=\gamma_{1,\max}$.

Before solving the CP optimization problem, to reduce the computational complexity, we first investigate the structure of the optimal solution of it. Let the singular-value decomposition of matrix $\left[f_{1},f_{2},f_{3}\right]$ be presented by

(12) $$\left[f_{1},f_{2},f_{3}\right]=U\Sigma V^{†},$$

where $U\in C^{K\times K}$, $\Sigma \in C^{K\times K}$ and $V\in C^{3\times K}$. U is an unitary matrix. $\Sigma =diag(\sigma_{1},\sigma_{2},...\sigma_{r})$, $\sigma_{i}>0$, i=1,2,...r are the positive singular values of $\left[f_{1},f_{2},f_{3}\right]$, and *r* is the rank of $\left[f_{1},f_{2},f_{3}\right]$. Then, r∈{1,2,3}. W.l.o.g., we assume $\sigma_{1}\geq \sigma_{2}\geq ...\geq \sigma_{r}$. Let $U=[U_{1},U_{2}]$, where $U_{1}$ is the matrix consisting of the first *r* columns of U. It is clear that $U_{1}⊥U_{2}$ i.e., $U_{1}^{†}U_{2}=0$. Proposition 1 is derived as follows.

**Proposition** **1.** *The optimal solutions of the CP problems (10) and problem (11) have the following stucture:*

(13) $$A=U_{1}^{*}{\mathrm{BU}}_{1}^{†}.$$

**Proof.**  See in Appendix B. □

It is known that the retransmitted signals vector can be expressed as

(14) $$\begin{array}{c} x_{R}={\mathrm{Af}}_{1}x_{1}+{\mathrm{Af}}_{2}x_{2}+{\mathrm{Az}}_{R}. \end{array}$$

According to the proof in Appendix B and the SVD of $\left[f_{1},f_{2},f_{3}\right]$, we find

(15) $$\begin{array}{c} {\mathrm{Af}}_{i}=U_{1}^{*}BU_{1}^{†}f_{i}+U_{2}^{*}DU_{1}^{†}f_{i}, \end{array}$$

and then

(16) $$\begin{array}{c} x_{R}=U_{1}^{*}BU_{1}^{†}\left(x_{1}f_{1}+x_{2}f_{2}\right)+U_{2}^{*}DU_{1}^{†}\left(x_{1}f_{1}+x_{2}f_{2}\right)+\left(U_{1}^{*}{\mathrm{BU}}_{1}^{†}+U_{1}^{*}{\mathrm{CU}}_{2}^{†}+U_{2}^{*}{\mathrm{DU}}_{1}^{†}+U_{2}^{*}{\mathrm{EU}}_{2}^{†}\right)z_{R}. \end{array}$$

We find that the components $U_{1}^{*}BU_{1}^{†}\left(x_{1}f_{1}+x_{2}f_{2}\right)$ and $U_{2}^{*}DU_{1}^{†}\left(x_{1}f_{1}+x_{2}f_{2}\right)$ are orthogonal. Additionally, the component $U_{2}^{*}DU_{1}^{†}\left(x_{1}f_{1}+x_{2}f_{2}\right)$ and $f_{3}$ are orthogonal. From a physical point of view, beamforming assigns the relay power as much as possible to the signal components that are non-orthogonal to the transmission direction. This means that D=0. For the noise components, $U_{2}^{*}{\mathrm{DU}}_{1}^{†}z_{R},U_{2}^{*}{\mathrm{EU}}_{2}^{†}z_{R}$ and $U_{1}^{*}{\mathrm{CU}}_{2}^{†}z_{R}$ should be eliminated employing beamforming, i.e., C=0, E=0. This result is the same as our discussion in Appendix B in mathematical optimization.

The optimal structure reduces the number of complex-valued design variables in the relay beamforming matrix from $K^{2}$ to $r^{2}$. Then, Equation (10) can be recast as follows:

(17) $$\begin{array}{cc} \max_{B}\;\; & \frac{{\left|g_{3}^{T}{\mathrm{Bg}}_{2}\right|}^{2}P_{2}}{\left|\right|g_{3}^{T}B{\left|\right|}^{2}+1},\\ s.t.\;\;\; & \frac{{\left|g_{3}^{T}{\mathrm{Bg}}_{1}\right|}^{2}P_{1}}{{\left|g_{3}^{T}{\mathrm{Bg}}_{2}\right|}^{2}P_{2}+\left|\right|g_{3}^{T}B{\left|\right|}^{2}+1}\geq \gamma_{1},\\ & \left|\right|{\mathrm{Bg}}_{1}{\left|\right|}^{2}P_{1}+\left|\right|{\mathrm{Bg}}_{2}{\left|\right|}^{2}P_{2}+\mathrm{tr}\left(BB^{†}\right)\leq P_{R,\max}, \end{array}$$

where $g_{i}=U_{1}^{†}f_{i}$, i=1,2. For the convenience of analysis, we modify the above problem as follows:

(18) $$\begin{array}{cc} \max_{b}\;\; & \frac{{\left|h_{2}^{T}b\right|}^{2}P_{2}}{\left|\right|H_{3}b{\left|\right|}^{2}+1},\\ s.t.\;\;\; & \frac{{\left|h_{1}^{T}b\right|}^{2}P_{1}}{{\left|h_{2}^{T}b\right|}^{2}P_{2}+\left|\right|H_{3}b{\left|\right|}^{2}+1}\geq \gamma_{1},\\ & b^{†}\Phi b\leq P_{R,\max}, \end{array}$$

where $b=\mathrm{vec}(B^{T})$, $h_{i}=\mathrm{vec}\left(g_{i}g_{3}^{T}\right)$, i=1,2, $H_{3}=g_{3}^{T}⊗I_{3}$, $\Theta =g_{1}g_{1}^{†}P_{1}+g_{2}g_{2}^{†}P_{2}+I_{3}$, and $\Phi =\mathrm{blkdiag}(\underset{r}{\underset{︸}{\Theta^{T},...,\Theta^{T}}})$. Obviously, there exists a matrix $\Psi =\Psi^{†}$ such that $\Phi =\Psi^{2}$.

### 3.2. Semi-Definite Programming-Based Approach

In this subsection, it is necessary to determine the range of $\gamma_{1}$, since an inappropriate $\gamma_{1}$ will lead to the feasible region of (10) being null. The maximum of $\gamma_{1}$ can be determined by the program as follows:

(19) $$\begin{array}{cc} \max_{b}\;\; & \frac{b^{†}h_{1}^{*}h_{1}^{T}bP_{1}}{b^{†}h_{2}^{*}h_{2}^{T}bP_{2}+b^{†}H_{3}^{†}H_{3}b+1},\\ s.t.\;\; & b^{†}\Phi b\leq P_{R,\max}. \end{array}$$

It is clear that the optimum of Equation (19) is attained when $b^{†}\Phi b=P_{R,\max}$ because the signals are transmitted in maximum relay power. Substituting this condition to the object function of (19), we have

(20) $$\begin{array}{cc} \max_{b}\;\; & \frac{b^{†}h_{1}^{*}h_{1}^{T}bP_{1}}{b^{†}h_{2}^{*}h_{2}^{T}bP_{2}+b^{†}H_{3}^{†}H_{3}b+b^{†}\Phi /P_{R,\max}b}. \end{array}$$

Before solving this program, we will introduce a lemma as follows.

**Lemma** **1.** *Given vectors a, $h\in C^{n\times 1}$ and a positive definite matrix $P\in C^{n\times n}$, and a function*

(21) $$\begin{array}{c} f\left(a\right)=\frac{a^{†}hh^{†}a}{a^{†}Pa}, \end{array}$$

*the maximum $h^{†}P^{-1}h$ is attained when $a=cP^{-1}h$, where c is an arbitrary complex constant.*

**Proof.**  See in Appendix C. □

Based on Lemma 1, $\gamma_{1,\max}=h_{1}^{T}{\left[h_{2}^{*}h_{2}^{T}P_{2}+H_{3}^{†}H_{3}+\Phi /P_{R,\max}\right]}^{-1}h_{1}^{*}P_{1}$ is attained at

(22) $$\begin{array}{c} b=c{\left[h_{2}^{*}h_{2}^{T}P_{2}+H_{3}^{†}H_{3}+\Phi /P_{R,\max}\right]}^{-1}h_{1}^{*}, \end{array}$$

where $c=e^{j\theta }\sqrt{P_{R,\max}}\left|\right|\Phi^{\frac{1}{2}}{\left[h_{2}^{*}h_{2}^{T}P_{2}+H_{3}^{†}H_{3}+\Phi /P_{R,\max}\right]}^{-1}h_{1}^{*}{\left|\right|}^{-1}$ for the power condition.

Next, we will develop several transformation tricks to reformulate the CP problem as an equivalent convex SDP problem, which can be efficiently solved. The problem (18) is first reformulated as

(23) $$\begin{array}{cc} \max_{b,u}\;\; & \frac{{\left|h_{2}^{T}b\right|}^{2}P_{2}}{u^{2}},\\ s.t.\;\;\; & \left|\right|H_{3}b{\left|\right|}^{2}+1=u^{2},\\ & \gamma_{1}({\left|h_{2}^{T}b\right|}^{2}P_{2}+\left|\right|H_{3}{b||}^{2}+1)-{\left|h_{1}^{T}b\right|}^{2}P_{1}\leq 0,\\ & b^{†}\Phi b\leq P_{R,\max}. \end{array}$$

Using the transformation tricks v=1/u and β=b/u, we have the following equivalent problem:

(24) $$\begin{array}{cc} \max_{\beta ,v}\;\; & {\left|h_{2}^{T}\beta \right|}^{2}P_{2},\\ s.t.\;\;\; & v^{2}=1-\left|\right|H_{3}\beta {\left|\right|}^{2},\\ & \gamma_{1}\left({\left|h_{2}^{T}\beta \right|}^{2}P_{2}+\left|\right|H_{3}\beta {\left|\right|}^{2}+v^{2}\right)-{\left|h_{1}^{T}\beta \right|}^{2}P_{1}\leq 0,\\ & \beta^{†}\Phi \beta -P_{R,\max}v^{2}\leq 0. \end{array}$$

Substituting the first constraint into the rest ones, we have

(25) $$\begin{array}{cc} \max_{\beta }\;\; & {\left|h_{2}^{T}\beta \right|}^{2}P_{2},\\ s.t.\;\;\; & \gamma_{1}\left({\left|h_{2}^{T}\beta \right|}^{2}P_{2}+1\right)-{\left|h_{1}^{T}\beta \right|}^{2}P_{1}\leq 0,\\ & \beta^{†}\Phi \beta +\left|\right|H_{3}\beta {\left|\right|}^{2}P_{R,\max}-P_{R,\max}\leq 0. \end{array}$$

Finally, using the transformation trick $X=\beta \beta^{†}$, (25) can be recast as

(26) $$\begin{array}{cc} \max_{X}\;\; & \mathrm{tr}\left(h_{2}^{*}h_{2}^{T}X\right)P_{2},\\ s.t.\;\;\; & \mathrm{tr}\left[(\gamma_{1}P_{2}h_{2}^{*}h_{2}^{T}-P_{1}h_{1}^{*}h_{1}^{T})X\right]+\gamma_{1}\leq 0,\\ & \mathrm{tr}\left[(\Phi +H_{3}^{†}H_{3}P_{R,\max})X\right]-P_{R,\max}\leq 0,\\ & X⪰0,\;\mathrm{rank}(X)=1. \end{array}$$

It is clear that the last rank-one constraint is non-convex. By applying the idea of the semi-definite relaxation (SDR) technique [20], the above problem can be relaxed to

(27) $$\begin{array}{cc} \max_{X}\;\; & \mathrm{tr}\left(h_{2}^{*}h_{2}^{T}X\right)P_{2},\\ s.t.\;\;\; & \mathrm{tr}\left[(\gamma_{1}P_{2}h_{2}^{*}h_{2}^{T}-P_{1}h_{1}^{*}h_{1}^{T})X\right]+\gamma_{1}\leq 0,\\ & \mathrm{tr}\left[(\Phi +H_{3}^{†}H_{3}P_{R,\max})X\right]-P_{R,\max}\leq 0,\\ & X⪰0, \end{array}$$

which is incorporated into a convex SDP problem [21] and thus can be efficiently solved via standard interior-point methods within polynomial time. Generally speaking, the resulting optimal solution may not lead to an optimal solution of (26) due to dropping the constraint rank(X)=1. Interestingly enough, it has been shown in [22] that, for the number of constraints less than three, the relaxed SDP problem always has a rank one solution, which is denoted by $X_{\mathrm{opt}}$. In other words, (26) and (27) are indeed equivalent. Consequently, we can obtain the optimal solution of (18), i.e., $b_{\mathrm{opt}}=\beta_{\mathrm{opt}}/v_{\mathrm{opt}}$, where $X_{\mathrm{opt}}=\beta_{\mathrm{opt}}\beta_{\mathrm{opt}}^{†}$ and $v_{\mathrm{opt}}=\sqrt{1-\left|\right|H_{3}\beta_{\mathrm{opt}}{\left|\right|}^{2}}$. It should be pointed out that the optimum and the optimal solution of (18) are with respect to $\gamma_{1}$. We need to take all $\gamma_{1}\in \left[0,\gamma_{1,\max}\right]$ to attain the hull of the achievable rate region.

## 4. Performance Analysis

To further investigate the performance of the proposed linear beamforming schemes, we study the maximum achievable individual and sum rates of cvx(R). We first analytically obtain the maximum individual rates of cvx(R) and the corresponding linear beamforming scheme. Then, we derive the maximum sum rate of cvx(R) in closed form and the corresponding linear beamforming scheme can be determined by solving a system of linear equations.

We consider first the maximum individual rates. Mathematically, the problem can be formulated as follows:

(28) $$\begin{array}{cc} \max_{A}\;\; & \frac{{\left|f_{3}^{T}{\mathrm{Af}}_{i}\right|}^{2}P_{i}}{\left|\right|f_{3}^{T}A{\left|\right|}^{2}+1},\\ s.t.\;\;\; & \left|\right|{\mathrm{Af}}_{1}{\left|\right|}^{2}P_{1}+\left|\right|{\mathrm{Af}}_{2}{\left|\right|}^{2}P_{2}+\mathrm{tr}\left({\mathrm{AA}}^{†}\right)\leq P_{R,\max}. \end{array}$$

According to Proposition 1 and the same argument above, it can be recast as

(29) $$\begin{array}{cc} \max_{b}\;\; & \frac{{\left|h_{i}^{T}b\right|}^{2}P_{i}}{\left|\right|H_{3}b{\left|\right|}^{2}+1},\\ s.t.\;\;\; & b^{†}\Phi b\leq P_{R,\max}. \end{array}$$

It is clear that the constraint holds with equality at the optimum, otherwise the optimal solution can always be scaled up yielding a larger objective value. Thus, substituting $b^{†}\Phi b/P_{R,\max}=1$ in the objective function, it can be formulated as follows:

(30) $$\begin{array}{c} \max_{b}\;\;\frac{b^{†}h_{i}^{*}h_{i}^{T}bP_{i}}{b^{†}\left(H_{3}^{†}H_{3}+\Phi /P_{R,\max}\right)b}. \end{array}$$

Using Lemma 1, we determine the maximum individual signal-to-noise ratios ${\mathrm{SNR}}_{i,\max}=h_{i}^{T}{\left(H_{3}^{†}H_{3}+\Phi /P_{R,\max}\right)}^{-1}h_{i}^{*}P_{i}$, i=1,2, which is attained at

(31) $$\begin{array}{c} b_{i,\mathrm{opt}}=c_{i}{\left(H_{3}^{†}H_{3}+\Phi /P_{R,\max}\right)}^{-1}h_{i}^{*}, \end{array}$$

where $c_{i}=e^{j\theta }\sqrt{P_{R,\max}}\left|\right|\Phi^{\frac{1}{2}}{\left(H_{3}^{†}H_{3}+\Phi /P_{R,\max}\right)}^{-1}h_{i}^{*}{\left|\right|}^{-1}$, which is chosen such that the constraint holds with equality. The maximum individual rates are easy to be determined $r_{i,\max}=\frac{1}{2}\log\left(1+{\mathrm{SNR}}_{i,\max}\right)$.

Next, we consider the maximum sum rate. Mathematically, the problem can be formulated as follows:

(32) $$\begin{array}{cc} \max_{A}\;\; & \frac{{\left|f_{3}^{T}{\mathrm{Af}}_{1}\right|}^{2}P_{1}+{\left|f_{3}^{T}{\mathrm{Af}}_{2}\right|}^{2}P_{2}}{\left|\right|f_{3}^{T}A{\left|\right|}^{2}+1},\\ s.t.\;\;\; & \left|\right|{\mathrm{Af}}_{1}{\left|\right|}^{2}P_{1}+\left|\right|{\mathrm{Af}}_{2}{\left|\right|}^{2}P_{2}+\mathrm{tr}\left({\mathrm{AA}}^{†}\right)\leq P_{R,\max}. \end{array}$$

Similarly, it can be recast as

(33) $$\begin{array}{cc} \max_{b}\;\; & \frac{{\left|h_{1}^{T}b\right|}^{2}P_{1}+{\left|h_{2}^{T}b\right|}^{2}P_{2}}{\left|\right|H_{3}b{\left|\right|}^{2}+1},\\ s.t.\;\;\; & b^{†}\Phi b\leq P_{R,\max}. \end{array}$$

For the same reason as in the previous case, the constraint holds with equality at the optimum. Thus, substituting $b^{†}\Phi b/P_{R,\max}=1$ in the objective function, it can be formulated as follows:

(34) $$\begin{array}{c} \max_{b}\;\;\frac{bh_{1}^{*}h_{1}^{T}bP_{1}+bh_{2}^{*}h_{2}^{T}bP_{2}}{b^{†}\left(H_{3}^{†}H_{3}+\Phi /P_{\max}\right)b}=\max_{b}\;\;\frac{b^{†}\left[\begin{array}{cc} h_{1}^{*} & h_{2}^{*} \end{array}\right]\left[\begin{array}{cc} P_{1} & 0\\ 0 & P_{2} \end{array}\right]\left[\begin{array}{c} h_{1}^{T}\\ h_{2}^{T} \end{array}\right]b}{b^{†}\left(H_{3}^{†}H_{3}+\Phi /P_{\max}\right)b}. \end{array}$$

Using Lemma 1, we will determine the maximum sum rate by the following Theorem 2.

**Theorem** **2.** *The maximum sum signal-to-noise ratio is denoted ${\mathrm{SNR}}_{\max}^{\mathrm{sum}}$, then*

(35) $$\begin{array}{c} {\mathrm{SNR}}_{\max}^{\mathrm{sum}}=\frac{\lambda_{1}+\lambda_{2}+\sqrt{{\left(\lambda_{1}+\lambda_{2}\right)}^{2}-4\lambda_{1}\lambda_{2}}}{2}, \end{array}$$

where $\lambda_{1}\lambda_{2}=P_{1}P_{2}\left[\left|\right|d_{1}{\left|\right|}^{2}\left|\right|d_{2}{\left|\right|}^{2}-{\left|d_{1}^{T}d_{2}^{*}\right|}^{2}\right]$, $\lambda_{1}+\lambda_{2}=\left|\right|d_{1}{\left|\right|}^{2}P_{1}+\left|\right|d_{2}{\left|\right|}^{2}P_{2}$ and ${\left[d_{1},d_{2}\right]}^{T}={\left[h_{1},h_{2}\right]}^{T}{\left(H_{3}^{†}H_{3}+\Phi /P_{R,\max}\right)}^{-\frac{1}{2}}$.

**Proof.**  See in Appendix D. □

The corresponding maximum sum rate is easily given by $r_{\mathrm{sum},\max}=\frac{1}{2}\log\left(1+{\mathrm{SNR}}_{\max}^{\mathrm{sum}}\right)$. The maximum is attained at $b_{\mathrm{sum},\mathrm{opt}},$ which can be determined by solving the following system of linear equations:

(36) $$\begin{array}{c} Mb_{\mathrm{sum},\mathrm{opt}}=0, \end{array}$$

where

(37) $$\begin{array}{cc} M= & {\left(H_{3}^{†}H_{3}+\Phi /P_{R,\max}\right)}^{-1}\left[h_{1}^{*},h_{2}^{*}\right]\mathrm{diag}\left(P_{1},P_{2}\right){\left[h_{1};h_{2}\right]}^{T}-\lambda_{\max}I, \end{array}$$

with an additional constraint that $b_{\mathrm{sum},\mathrm{opt}}^{†}\Phi b_{\mathrm{sum},\mathrm{opt}}=P_{R,\max}$. Through the mathematical analysis above, we obtain the two maximum individual rates $r_{1,\max}$, $r_{2,\max}$ and the maximum sum rate $r_{\mathrm{sum},\max}$. These three values will determine the bound of the theoretical rate region easily, but we can not determine the beamforming matrices corresponding to all of the points on the bound of the rate region.

Next, we will discuss whether the outer bound determined by these three maximum rates $C_{1}\left(A_{\mathrm{opt},1}\right)=r_{1,\max}$, $C_{2}\left(A_{\mathrm{opt},2}\right)=r_{2,\max}$ and $C_{\mathrm{sum}}\left(A_{\mathrm{opt},1}\right)=r_{\mathrm{sum},\max}$ is tight. We know if this outer bound is tight, for the “upper corner” of the rare region R(A), there exists a beamforming matrix $\hat{A}\in \Omega$, such that

(38) $$\begin{array}{cc} R_{1}^{\mathrm{up}}\left(\hat{A}\right) & =\max_{A\in \Omega }\frac{1}{2}\log\left(1+\frac{|f_{3}^{T}{\mathrm{Af}}_{1}{|}^{2}P_{1}}{|f_{3}^{T}{\mathrm{Af}}_{2}{|}^{2}P_{2}+\left|\right|f_{3}^{T}A{\left|\right|}^{2}+1}\right)=\frac{1}{2}\log\left(1+\gamma_{1,\max}\right), \end{array}$$

(39) $$\begin{array}{cc} R_{2}^{\mathrm{up}}\left(\hat{A}\right) & =\max_{A\in \Omega }\frac{1}{2}\log\left(1+\frac{|f_{3}^{T}{\mathrm{Af}}_{2}{|}^{2}P_{2}}{\left|\right|f_{3}^{T}A{\left|\right|}^{2}+1}\right)=r_{2,\max}, \end{array}$$

all hold. According to the previous analysis, the condition achieving $\gamma_{1,\max}$ is

(40) $$\begin{array}{c} b=c{\left[h_{2}^{*}h_{2}^{T}P_{2}+H_{3}^{†}H_{3}+\Phi /P_{R,\max}\right]}^{-1}h_{1}^{*}, \end{array}$$

where $c=e^{j\theta }\sqrt{P_{R,\max}}\left|\right|\Phi^{\frac{1}{2}}{\left[h_{2}^{*}h_{2}^{T}P_{2}+H_{3}^{†}H_{3}+\Phi /P_{R,\max}\right]}^{-1}h_{1}^{*}{\left|\right|}^{-1}$ . The condition achieving $r_{2,\max}$ is

(41) $$\begin{array}{c} b=\hat{c}{\left(H_{3}^{†}H_{3}+\Phi /P_{R,\max}\right)}^{-1}h_{2}^{*}, \end{array}$$

where $\hat{c}=e^{j\theta }\sqrt{P_{R,\max}}\left|\right|\Phi^{\frac{1}{2}}{\left(H_{3}^{†}H_{3}+\Phi /P_{R,\max}\right)}^{-1}h_{2}^{*}{\left|\right|}^{-1}$ . Obviously, (40) and (41) can not be established simultaneously. Therefore, this outer bound is not tight.

## 5. Numerical Results

In this section, we show some numerical results to quantify the achievable rate regions of a dual-hop MARN with linear beamforming, and analyze the transmission performance under different antenna numbers and relay schemes. All simulations are performed in MATLAB r2010a (MathWorks, Natick, MA, USA). We use CVX toolbox [23] to solve the SDP problems. We assume that the power budgets of the two sources are $P_{1}=P_{2}=3\;\mathrm{dBW}$, and the relay is equipped with *K* antennas having a relay power budget $P_{R}$. The channel coefficients are generated as independent complex Gaussian random variables with the distribution CN(0,1). We randomly generate a set of channel vectors $f_{1},f_{2}$ and $f_{3}$ as follows:

$$\begin{array}{cc} f_{1} & =[0.091-0.858i,0.464-0.933i,-0.826+0.658i,-0.326+0.008i,-0.186-0.456i],\\ f_{2} & =[0.570+1.046i,0.164+0.805i,-0.700-0.484i,0.947-0.914i,0.205-0.052i],\\ f_{3} & =[-0.234-0.600i,-0.597-0.174i,0.352+0.469i,1.053-0.604i,-0.386-0.849i]. \end{array}$$

**Figure 5:**
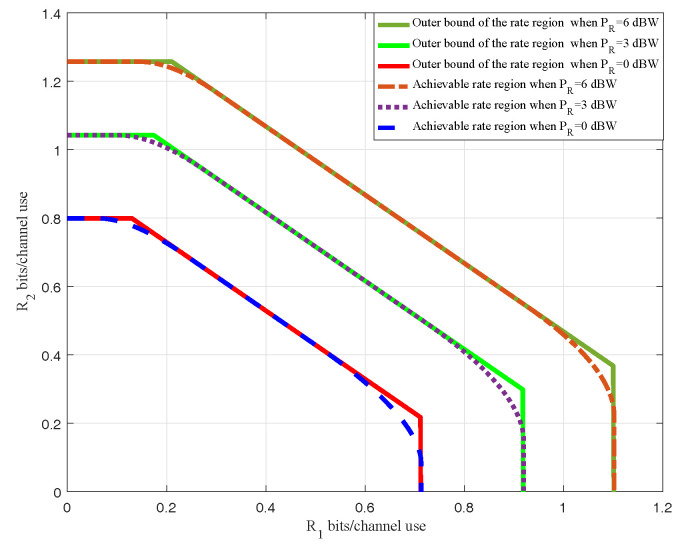
Achievable rate region and Outer bounds.

First, setting the channel vector $f_{1},f_{2}$ and $f_{3}$ above and the number of antennas *K* at relay. We show the achievable rate regions versus different relay power budgets $P_{R}=0,3,6\;\mathrm{dBW}$ in Figure 5. Meanwhile, according to the three rates $r_{1,\max},r_{2,\max}$ and $r_{\mathrm{sum},\max}$ obtained employing the method in Section 4, we determine an outer bound of the achievable rate region R. The simulation results show that the achievable rate regions obtained by using our scheme almost coincides with the corresponding outer bounds except the corners. It validates the conclusion obtained through analysis in Section 4 that this outer bound is not tight. It is observed that the achievable rate region with $P_{R,\max}=0$ dBW is the interior of the achievable rate region with $P_{R,\max}=3,6$ dBW. The achievable rate region expands as the relay power budget $P_{R,\max}$ increases. It is in conformity with the reality that increasing the power budget of relay antennas always leads to better transmission performance.

**Figure 6:**
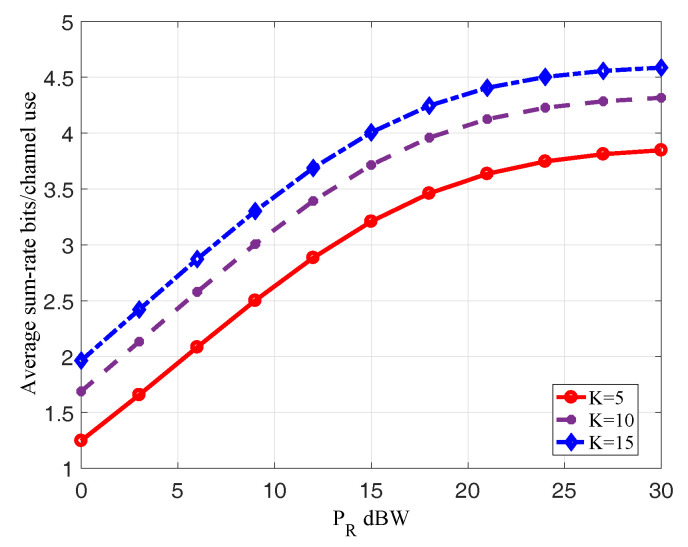
Average Sum-rate when K=5,10,15 versus the $P_{R}$.

In Figure 6, we show the average sum-rates corresponding to the optimal beamforming matrices versus different relay power budget $P_{R}=0,3,6,...,30\;\mathrm{dBW}$ when K=5,10,15 over 5000 channel realization. Comparing these three curves, we find the average sum-rates increase with the increase of *K*. It is in conformity with the reality that increasing the number of antennas *K* can increase diversity gain to improve system transmission performance. In addition, the average sum-rates increase with the increase of the relay power budget $P_{R}$, which is consistent with the result in Figure 5.

To further illustrate the system performance exploiting the scheme proposed in this paper, we show the average sum-rates versus different relay power budgets $P_{R}=0,3,6,...,30\;\mathrm{dBW}$ comparing with other several relay schemes that are usually applied in practice.

1. Direct relaying, where the relay beamforming matrix is in the form of A=αI and $\alpha =\sqrt{\frac{P_{R,\max}}{\left|\right|f_{1}{\left|\right|}^{2}P_{1}+\left|\right|f_{2}{\left|\right|}^{2}P_{2}+K}}$. It is a scheme to uniformly amplify all the signals of antennas at relay. In essence, it is not a beamforming scheme but a simple AF scheme.
2. Alternative relaying, which is a TDMA scheme. It requires four time slots to complete the communication between the two sources $S_{1},S_{2}$ and the destination. We assume that the communication between source $S_{1}$ and destination is completed in the first and second time slots, while communication between source $S_{2}$ and destination is completed in the other two time slots. The corresponding relay beamforming matrices are given in [6].
3. Optimal diagonal beamforming, which is a beamforming scheme that the signals of the different antennas can not be superimposed. The corresponding relay beamforming matrices are diagonal matrices, which is an optimal form of the direct relaying scheme.

**Figure 7:**
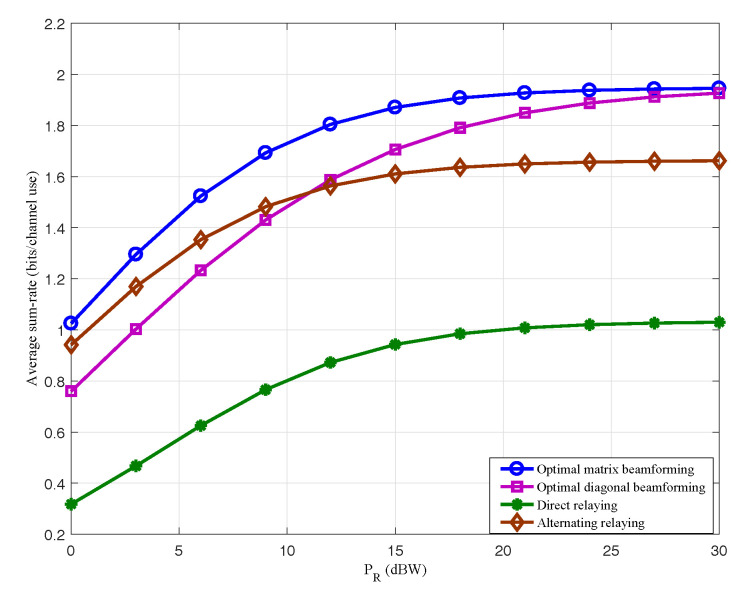
Average Sum-rates of different relaying schemes.

It is observed in Figure 7 that the optimal linear beamforming scheme significantly outperforms the other three relay schems.

## 6. Conclusions

In this paper, we investigate a dual-hop MARN consisting of two single-antenna sources, a single-antenna destination and a helping multi-antenna relay. The relay assists the communication between the sources and destination using an ANC-based linear beamforming scheme. We characterize the achievable rate region and acquire the corresponding beamforming schemes. In addition, we analyze the optimal linear beamforming schemes for the individual rates and sum-rate, and derive the mathematical closed forms of maximum individual rate and sum-rate. However, in order to implement the linear beamforming scheme, the CSI of the sources and destination should be acquired at relay. The SCI acquiring technique and feedback mechanism are used to provide the relay with all of CSIs, yielding higher overhead and complexity, which is the main challenge for designing a beamforming scheme. Our future work will be related to the imperfect stochastic or deterministic CSI model.

## Appendix A. Proof of Theorem 1

**Proof.** Since $(r_{1},r_{2})\in R$, we assume that $\left(r_{1},r_{2}\right)\in R\left(A\right)$, $r_{2}=C_{2}\left(A\right)$ and $r_{1}+r_{2}\leq C_{\mathrm{sum}}\left(A\right)$ for some A∈Ω. $A_{o}\left(r_{1}\right)$ is the optimal solution of problem (10) satisfying $A_{o}\left(r_{1}\right)\in \Omega$ and the condition as follows:

(A1) $$\begin{array}{c} \frac{|f_{3}^{T}A_{o}\left(r_{1}\right)f_{1}{|}^{2}P_{1}}{|f_{3}^{T}A_{o}\left(r_{1}\right)f_{2}{|}^{2}P_{2}+\left|\right|f_{3}^{T}A_{o}\left(r_{1}\right){\left|\right|}^{2}+1}\geq \gamma_{1}. \end{array}$$

According to (8),

(A2) $$\begin{array}{c} R_{1}^{\mathrm{up}}\left(A_{o}\left(r_{1}\right)\right)=\frac{1}{2}\log\left(1+\frac{|f_{3}^{T}A_{o}\left(r_{1}\right)f_{1}{|}^{2}P_{1}}{|f_{3}^{T}A_{o}\left(r_{1}\right)f_{2}{|}^{2}P_{2}+\left|\right|f_{3}^{T}A_{o}\left(r_{1}\right){\left|\right|}^{2}+1}\right)\geq \frac{1}{2}\log\left(1+\gamma_{1}\right)=r_{1}. \end{array}$$

For $R_{1}^{\mathrm{up}}\left(A_{o}\left(r_{1}\right)\right)\leq R_{1}^{\mathrm{low}}\left(A_{o}\left(r_{1}\right)\right)$, then, $r_{1}\leq R_{1}^{\mathrm{low}}\left(A_{o}\left(r_{1}\right)\right)=C_{1}\left(A_{o}\left(r_{1}\right)\right)$. Considering the “up corner” in R(A), we have

(A3) $$\begin{array}{c} R_{1}^{\mathrm{up}}\left(A\right)=C_{\mathrm{sum}}\left(A\right)-R_{2}^{\mathrm{up}}\left(A\right)=C_{\mathrm{sum}}\left(A\right)-C_{2}\left(A\right)\geq r_{1}+r_{2}-C_{2}\left(A\right)=r_{1}. \end{array}$$

Then, the condition $\frac{|f_{3}^{T}Af_{1}{|}^{2}P_{1}}{|f_{3}^{T}Af_{2}{|}^{2}P_{2}+\left|\right|f_{3}^{T}A{\left|\right|}^{2}+1}\geq \gamma_{1}$ holds. From problem (10), we have

$$\begin{array}{c} C_{2}\left(A_{o}\left(r_{1}\right)\right)=R_{2}^{\mathrm{up}}\left(A_{o}\left(r_{1}\right)\right)=\frac{1}{2}\log\left(1+\frac{|f_{3}^{T}A_{o}\left(r_{1}\right)f_{2}{|}^{2}P_{2}}{\left|\right|f_{3}^{T}A_{o}\left(r_{1}\right){\left|\right|}^{2}+1}\right) \end{array}$$

(A4) $$\begin{array}{c} \geq \frac{1}{2}\log\left(1+\frac{|f_{3}^{T}Af_{2}{|}^{2}P_{2}}{\left|\right|f_{3}^{T}A{\left|\right|}^{2}+1}\right)=C_{2}\left(A\right)=r_{2}, \end{array}$$

(A5) $$\begin{array}{c} r_{1}+r_{2}\leq R_{1}^{\mathrm{up}}\left(A_{o}\left(r_{1}\right)\right)+R_{2}^{\mathrm{up}}\left(A_{o}\left(r_{1}\right)\right)=C_{\mathrm{sum}}\left(A_{o}\left(r_{1}\right)\right). \end{array}$$

As a result, from (6), we can conclude that $\left(r_{1},r_{2}\right)\in R\left(A_{o}\left(r_{1}\right)\right)$. □

## Appendix B. Proof of Proposition 1

**Proof.** Since singular value decomposition $\left[f_{1},f_{2},f_{3}\right]=U\Sigma V^{†}$, we know that $U=[U_{1},U_{2}]$, where $U_{1}\in C^{K\times r}$ is the first *r* columns of U. Since U is a unitary matrix, it is clear that $U_{1}^{†}U_{2}=0$, $U_{1}^{†}U_{1}=I_{r}$, and $U_{2}^{†}U_{2}=I_{K-r}$. W.l.o.g., the AF beamforming matrix A can be written as follows:

(A6) $$\begin{array}{cc} A & =U^{*}\left[\begin{array}{cc} B & C\\ D & E \end{array}\right]U^{†}=\left[U_{1}^{*},U_{2}^{*}\right]\left[\begin{array}{cc} B & C\\ D & E \end{array}\right]\left[\begin{array}{c} U_{1}^{†}\\ U_{2}^{†} \end{array}\right]=U_{1}^{*}{\mathrm{BU}}_{1}^{†}+U_{1}^{*}{\mathrm{CU}}_{2}^{†}+U_{2}^{*}{\mathrm{DU}}_{1}^{†}+U_{2}^{*}{\mathrm{EU}}_{2}^{†}, \end{array}$$

where $B\in C^{r\times r}$, $C\in C^{r\times (K-r)}$, $D\in C^{(K-r)\times r}$ and $E\in C^{\left(K-r)\times (K-r\right)}$ are arbitrarily matrices. Additionally,

(A7) $$\begin{array}{c} U^{†}\left[f_{1},f_{2},f_{3}\right]=U^{†}U\Sigma V^{†}=I_{K}\left[\begin{array}{c} \hat{\Sigma }\\ 0 \end{array}\right]V^{†}=\left[\begin{array}{c} \hat{\Sigma }V^{†}\\ 0 \end{array}\right], \end{array}$$

where $\hat{\Sigma }\in C^{r\times K}$ is the first *r* rows of Σ. Furthermore, we find,

(A8) $$\begin{array}{c} U^{†}\left[f_{1},f_{2},f_{3}\right]=\left[\begin{array}{c} U_{1}^{†}\\ U_{2}^{†} \end{array}\right]\left[f_{1},f_{2},f_{3}\right]=\left[\begin{array}{ccc} U_{1}^{†}f_{1} & U_{1}^{†}f_{2} & U_{1}^{†}f_{3}\\ U_{2}^{†}f_{1} & U_{2}^{†}f_{2} & U_{2}^{†}f_{3} \end{array}\right]. \end{array}$$

Comparing (A7) with (A8), we find $U_{2}^{†}f_{i}=0$, i=1,2,3. Then,

(A9) $$\begin{array}{cc} |f_{3}^{T}Af_{i}{|}^{2} & =|f_{3}^{T}U_{1}^{*}BU_{1}^{†}f_{i}+f_{3}^{T}U_{1}^{*}CU_{2}^{†}f_{i}+f_{3}^{T}U_{2}^{*}DU_{1}^{†}f_{i}+f_{3}^{T}U_{2}^{*}EU_{2}^{†}f_{i}{|}^{2}={\left|f_{3}^{T}U_{1}^{*}BU_{1}^{†}f_{i}\right|}^{2}, \end{array}$$

which is independent of C, D, and E,

(A10) $$\begin{array}{cc} \left|\right|f_{3}^{T}A{\left|\right|}^{2} & =\left|\right|f_{3}^{T}U_{1}^{*}BU_{1}^{†}+f_{3}^{T}U_{1}^{*}CU_{2}^{†}+f_{3}^{T}U_{2}^{*}DU_{1}^{†}+f_{3}^{T}U_{2}^{*}EU_{2}^{†}{\left|\right|}^{2}=\left|\right|f_{3}^{T}U_{1}^{*}BU_{1}^{†}+f_{3}^{T}U_{1}^{*}CU_{2}^{†}{\left|\right|}^{2}, \end{array}$$

(A11) $$\begin{array}{cc} ||Af_{i}{\left|\right|}^{2} & =\left|\right|U_{1}^{*}BU_{1}^{†}f_{i}+U_{1}^{*}CU_{2}^{†}f_{i}+U_{2}^{*}DU_{1}^{†}f_{i}+U_{2}^{*}EU_{2}^{†}f_{i}{\left|\right|}^{2}=\left|\right|U_{1}^{*}BU_{1}^{†}f_{i}+U_{2}^{*}DU_{1}^{†}f_{i}{\left|\right|}^{2}. \end{array}$$

Since $U_{1}^{†}U_{2}=0$, $U_{1}^{†}U_{1}=I_{r}$ and $U_{2}^{†}U_{2}=I_{K-r}$, it is easy to check that

(A12) $$\begin{array}{cc} \left|\right|f_{3}^{T}A{\left|\right|}^{2} & =\left|\right|f_{3}^{T}U_{1}^{*}B{\left|\right|}^{2}+\left|\right|f_{3}^{T}U_{1}^{*}C{\left|\right|}^{2}, \end{array}$$

(A13) $$\begin{array}{c} \left|\right|{\mathrm{Af}}_{i}{\left|\right|}^{2}=\left|\right|{\mathrm{BU}}_{1}^{†}f_{i}{\left|\right|}^{2}+\left|\right|{\mathrm{DU}}_{1}^{†}f_{i}{\left|\right|}^{2}, \end{array}$$

and

(A14) $$\mathrm{tr}\left({\mathrm{AA}}^{†}\right)=\mathrm{tr}\left({\mathrm{BB}}^{†}\right)+\mathrm{tr}\left({\mathrm{CC}}^{†}\right)+\mathrm{tr}\left({\mathrm{DD}}^{†}\right)+\mathrm{tr}\left({\mathrm{EE}}^{†}\right).$$

Substituting the corresponding equations above into the CP program (10), the program can be rewritten as follows:

(A15) $$\begin{array}{cc} & \max_{B,C}\;\;\frac{|f_{3}^{T}U_{1}^{*}BU_{1}^{†}f_{2}{|}^{2}P_{2}}{\left|\right|f_{3}^{T}U_{1}^{*}{B||}^{2}+\left|\right|f_{3}^{T}U_{1}^{*}C{\left|\right|}^{2}+1},\\ s.t\;\; & \frac{|f_{3}^{T}U_{1}^{*}BU_{1}^{†}f_{1}{|}^{2}P_{1}}{|f_{3}^{T}U_{1}^{*}BU_{1}^{†}f_{2}{|}^{2}P_{2}+\left|\right|f_{3}^{T}U_{1}^{*}{B||}^{2}+\left|\right|f_{3}^{T}U_{1}^{*}C{\left|\right|}^{2}+1}\geq \gamma_{1},\\ & ||BU_{1}^{*}f_{1}{\left|\right|}^{2}P_{1}+||BU_{1}^{*}f_{2}{\left|\right|}^{2}P_{2}+\mathrm{tr}\left(BB^{†}\right)+\mathrm{tr}\left(CC^{†}\right)\\ & \leq P_{R,\max}-||DU_{1}^{*}f_{1}{\left|\right|}^{2}P_{1}-||DU_{1}^{*}f_{2}{\left|\right|}^{2}P_{2}\\ & -\mathrm{tr}\left(DD^{†}\right)-\mathrm{tr}\left(EE^{†}\right). \end{array}$$

Obviously, the object function is independent of D,E. The feasible region will be expanded when D=0 and E=0. Then, the maximum of (A15) is attained when C=0. Thus, it can be concluded that the matrices C,D and E are all set to zero at the optimal. □

## Appendix C. Proof of Lemma 1

**Proof.** For P is a positive definite matrix, there exists an invertible matrix T satisfying $P=T^{†}T$. Setting d=Ta, and then substituting $a=T^{-1}d$ in function (21), we have

(A16) $$\begin{array}{c} \frac{d^{†}{\left(T^{-1}\right)}^{†}hh^{†}T^{-1}d}{d^{†}d}, \end{array}$$

which is a standard Rayleight–Ritz quotient form [24], and the maximum is the maximal eigenvalue of the matrix ${\left(T^{-1}\right)}^{†}hh^{†}T^{-1}$. Obviously, $\mathrm{rank}({\left(T^{-1}\right)}^{†}hh^{†}T^{-1})=1$ and there exists only one non-zero eigenvalue λ, which is equal to

(A17) $$\begin{array}{cc} \lambda & =h^{†}T^{-1}{\left(T^{†}\right)}^{-1}h=h^{†}{\left(T^{†}T\right)}^{-1}h=h^{†}P^{-1}h>0. \end{array}$$

Apparently, (A16) equals λ when d is the eigenvector of the matrix ${\left(T^{-1}\right)}^{†}hh^{†}T^{-1}$. We know that d can be expressed as

(A18) $$\begin{array}{c} d=c{\left(T^{†}\right)}^{-1}h, \end{array}$$

where *c* is an arbitrary complex constant, and then it is deduced that

(A19) $$\begin{array}{cc} a & =cT^{-1}{\left(T^{†}\right)}^{-1}h=c{\left(T^{†}T\right)}^{-1}h=cP^{-1}h. \end{array}$$

□

## Appendix D. Proof of Theorem 2

**Proof.** Obviously, $H_{3}^{†}H_{3}+\Phi /P_{R,\max}$ is a positive definite matrix, for notation simplicity, we denote

(A20) $$\begin{array}{c} \Lambda ={\left(H_{3}^{†}H_{3}+\Phi /P_{R,\max}\right)}^{-\frac{1}{2}}. \end{array}$$

Then, $\Lambda =\Lambda^{†}$, setting $\left[\begin{array}{c} d_{1}^{T}\\ d_{2}^{T} \end{array}\right]=\left[\begin{array}{c} h_{1}^{T}\\ h_{2}^{T} \end{array}\right]\Lambda$ and x=Λb. The objective function can be reformulated as

(A21) $$\begin{array}{c} \max_{x}\;\;\frac{x^{†}\left[d_{1}^{*},d_{2}^{*}\right]\left[\begin{array}{cc} P_{1} & 0\\ 0 & P_{2} \end{array}\right]\left[\begin{array}{c} d_{1}^{T}\\ d_{2}^{T} \end{array}\right]x}{x^{†}x}=\max_{x}\;\;\frac{x^{†}\left[d_{1}^{*},d_{2}^{*}\right]\mathrm{diag}\left(P_{1},P_{2}\right){\left[d_{1};d_{2}\right]}^{T}x}{x^{†}x}. \end{array}$$

Since the maximum value of the above function is independent of ||x||, the constraint can always be satisfied and thus be omitted. Note that the maximum value of the Rayleigh–Ritz quotient [24] is equal to the proper generalized eigenvalue. Therefore, for the above function, the maximum value is equal to the proper eigenvalue of matrix $\left[d_{1}^{*},d_{2}^{*}\right]\mathrm{diag}\left(P_{1},P_{2}\right){\left[d_{1};d_{2}\right]}^{T}$. By Sylvester’s Theorem, the matrix

(A22) $$\begin{array}{cc} \Psi & ={\left[d_{1};d_{2}\right]}^{T}\left[d_{1}^{*},d_{2}^{*}\right]\mathrm{diag}\left(P_{1},P_{2}\right)=\left[\begin{array}{cc} \left|\right|d_{1}{\left|\right|}^{2}P_{1} & d_{1}^{T}d_{2}^{*}P_{2}\\ d_{2}^{T}d_{1}^{*}P_{1} & \left|\right|d_{2}{\left|\right|}^{2}P_{2} \end{array}\right] \end{array}$$

has exactly the same non-zero eigenvalues as $\left[d_{1}^{*},d_{2}^{*}\right]\mathrm{diag}\left(P_{1},P_{2}\right){\left[d_{1};d_{2}\right]}^{T}$. Denote $\lambda_{1}$ and $\lambda_{2}$ the eigenvalues of the above matrix. Then, we have the following relationships:

(A23) $$\begin{array}{c} \lambda_{1}\lambda_{2}=\det\left(\Psi \right)=P_{1}P_{2}\left[\left|\right|d_{1}{\left|\right|}^{2},||d_{2}{\left|\right|}^{2}-{\left|d_{1}^{T}d_{2}^{*}\right|}^{2}\right], \end{array}$$

(A24) $$\begin{array}{c} \lambda_{1}+\lambda_{2}=\mathrm{tr}\left(\Psi \right)=\left|\right|d_{1}{\left|\right|}^{2}P_{1}+\left|\right|d_{2}{\left|\right|}^{2}P_{2}. \end{array}$$

Consequently, the maximum eigenvalue $\lambda_{\max}$ of Ψ is given as follows:

(A25) $$\lambda_{\max}=\frac{\lambda_{1}+\lambda_{2}+\sqrt{{\left(\lambda_{1}+\lambda_{2}\right)}^{2}-4\lambda_{1}\lambda_{2}}}{2}.$$

□
